# The Role of Community Pharmacists in Travel Health and Vaccination in Switzerland

**DOI:** 10.3390/pharmacy6040125

**Published:** 2018-11-29

**Authors:** Claudine Leuthold, Olivier Bugnon, Jérôme Berger

**Affiliations:** 1 PharmaSuisse, the Swiss Association of Pharmacists, CH-3097 Bern-Liebefeld, Switzerland; claudine.leuthold@pharmasuisse.org; 2 Community Pharmacy Centre, Department of Ambulatory Care and Community Medicine, University of Lausanne, CH-1011 Lausanne, Switzerland; olivier.bugnon@hospvd.ch; 3 Community Pharmacy Practice Research, School of pharmaceutical sciences, University of Geneva, CH-1206 Genève, Switzerland; 4 Community Pharmacy Practice Research, School of pharmaceutical sciences, University of Lausanne, CH-1206 Genève, Switzerland

**Keywords:** travel medicine, pharmacy, community, travel, practice, vaccination, Switzerland, education

## Abstract

This review presents the Swiss strategy initiated over the last several years to implement vaccination by community pharmacists. National health authorities aimed to integrate community pharmacists in the National Vaccination Strategy (NVS) in order to increase the vaccination rate in the Swiss population. To support this aim, universities and the Swiss Association of Pharmacists developed pre- and post-graduate education programmes on vaccination for pharmacists. Finally, each Swiss canton (sovereign for health-related aspects) set proper regulations to authorize pharmacists to vaccinate and to determine which vaccines could be administered. As of September 2018, 19 cantons (out of 26) had authorized influenza vaccinations under the sole responsibility of an accredited community pharmacist. Additional vaccinations were available in 13 cantons (e.g., tick-borne encephalitis or hepatitis A, B, or A and B). Such implementation in other countries should follow a similar top-down (following a national strategy to improve vaccination coverage) and stepwise (starting with influenza to demonstrate the competencies of community pharmacists) strategy, supported by the development of research, education and accreditation. The development of health advice related to travels in community pharmacies should follow the same development in Switzerland. Currently, it offers the opportunity for strengthening travellers’ safety, beyond vaccination issues.

## 1. Introduction

Until 2015, a prescription was required to authorize Swiss community pharmacists to supply vaccines, which were administered by other health professionals. This review presents the legal and practice changes that occurred in the last years to implement vaccination in community pharmacies and to include community pharmacists in the National Vaccination Strategy (NVS) from Swiss health authorities. This article also summarizes the activities, education, current situation, and legal frame of Swiss community pharmacists in the field of travel health and vaccination. It is based on reports from the heath authorities and from the national association of pharmacists (pharmaSuisse). Finally, it proposes future developments to strengthen the roles of community pharmacists in health services related to travel and vaccination.

## 2. Community Pharmacists and Travel Health

In Switzerland, any pharmacist has the basic skills to provide specific advice related to health promotion while traveling, in accordance with the responsibilities assigned by the Federal Law on Medical Professions [1]. This is an important activity, as 8.4 million Swiss citizens travel extensively (approximately 1.8 million trips outside Europe in 2017) [2]. This advice includes, for example, safe sex, sun protection, mosquito bite prevention, water purification or motion sickness treatment. To support this activity, pharmacists can provide sanitary materials, pharmacy kits or non-prescription medicines. In addition, pharmacists have to ensure the responsible use of antimalarial and anti-infective medicines, as well as vaccines, that they deliver under medical prescription. Their recommendations related to malaria and infectious disease risks are based on those issued by the Travel Medicine Centre of the University of Zurich and the Swiss Tropical and Public Health Institute of Basel. These are published by the Federal Office of Public Health (FOPH) [3], which is responsible for public health in Switzerland and develops Switzerland’s health policies and contributes to ensuring that the country has an efficient and affordable healthcare system [4]. The practice recommendations issued by the FOPH are disseminated by two websites that can be used by community pharmacists, one for the public (www.safetravel.ch) and one for health professional (www.tropimed.ch). These websites display useful information related to travel health, as well as official recommendations adapted to the target audiences, completed with additional related information and maps.

## 3. Community Pharmacists and Vaccination

In addition to the activities related to health promotion while traveling that can be performed on a national level by any community pharmacist, some accredited pharmacists are authorized to administer some vaccines, e.g., to people seeking advice before travelling abroad (see Table 1). This accreditation depends on the canton where the community pharmacists are practising. Indeed, each Swiss canton is sovereign for health-related aspects: community pharmacists can be authorized or not to vaccinate and the list of vaccines they can administer varies among cantons. To be authorized to vaccinate in a canton where this practice is possible, a community pharmacist must undergo post-graduate education.

The FOPH integrated pharmacists as potential actors and partners for vaccination in the NVS that was initiated from 2012 to 2017 [6]. This integration was based on the assessment of the influenza national vaccination campaigns performed in 2008 to 2012, which did not include community pharmacists [7]. This assessment showed that most of the goals of the campaign were not reached because of three main causes: (1). the vaccination rate of the various target groups decreased over the time; (2) the health professionals (mainly physicians) did not implement vaccinations in their daily practice; and (3) the “multiplier groups” did not include enough health professionals (the FOPH defined “multiplier groups” as physicians, cantonal health authorities, or media in charge of supporting and disseminating the health authorities’ messages regarding vaccination). The community pharmacists were identified as able to reach the “healthy” population that had no regular contact with a general practitioner (GP). For example, in 2012, 34% of Swiss citizens above 15 years old declared to have had no appointment with a GP in the previous 12 months [8]. In addition, community pharmacists were considered as a potential “multiplier group” to increase the vaccination coverage in the Swiss population. Indeed, there is approximately one community pharmacy for every 4700 people in Switzerland [9]. Another element that advocated towards the inclusion of community pharmacists in the NVS was that the FOPH wanted to promote the use of the electronic vaccination plan (www.myvaccines.ch); thirty percent of community pharmacies were already subscribers of this website [10].

Based on the aim of the FOPH to integrate community pharmacists in the NVS and on foreign experiences of vaccination services in community pharmacies, the Swiss Association of Pharmacists (pharmaSuisse) initiated a post-graduate educational programme to train and accredit community pharmacists for vaccination. Based on American and Portuguese experiences, a Swiss post-graduate training certificate named (in French) “Certificat de formation complémentaire FPH Vaccination et prélèvements sanguins (Foederatio Pharmaceutica Helvetiae)” [11] was created in 2011. The first community pharmacists were accredited in 2012.

Following the recommendations of the FOPH to encourage vaccination in community pharmacies and the post-graduate training in vaccination for community pharmacists, some cantons began to authorize vaccination by trained and accredited community pharmacists in 2015. This required changes in the Swiss laws to allow community pharmacists to administer a vaccine without a prior medical prescription. Then, each canton had to establish proper regulations to determine which vaccines could be administered and which facilities were required on the premises of community pharmacies to perform vaccination.

As of September 2018, the situation in the 26 Swiss cantons was as follows: six cantons (Aargau, Appenzell Inner-Rhodes, Appenzell Outer Rhodes, Glarus, Obwalden, and Uri) had not yet authorized vaccination in community pharmacies, one canton (Ticino) had authorized vaccination only when prescribed by a physician, and 19 cantons had authorized vaccinations under the sole responsibility of an accredited community pharmacist (see Table 1). In the cantons that authorized vaccinations by accredited community pharmacists, influenza vaccination was the first to be available, and it was followed by other vaccinations in 16 cantons. Age limits have to be considered for vaccination in community pharmacies; this is only approved for people older than 16 years old (18 years old in Basel-Stadt and Basel-Landschaft), and two cantons (Geneva and Valais) do not permit vaccination of people over 65 years old. Currently, more than 1400 community pharmacists (out of 5300) are accredited and approximately 700 pharmacies (out of 1800) are available for vaccination [5].

## 4. Education of Community Pharmacists related to Travel Health and Vaccination

Three different universities (Basel, Geneva, and Zürich) offer a full curriculum for pharmacy students in Switzerland. Pre-graduate training objectives are defined at the national level by FOPH in concordance with the Federal Law on Medical Professions [12]. The national vaccination schemes as well as the responsible use of the vaccines registered on the Swiss market are included in the objectives. This is the common minimum base related to vaccination that has to be taught in each university. Beside this, each university completes its lessons and learning objectives according to the needs of local community pharmacists. For example, at the University of Geneva, 4 h on health advices related to travel in community pharmacy (e.g., malaria prevention or travelling with medicines) and 4 h on vaccination (e.g., vaccination schemes, vaccination booklet, and advice on vaccination in community pharmacy) are taught to master students in pharmacy. In addition, pre-graduate courses are currently reviewed according to revised national learning objectives for community pharmacists. Hence, injection and blood sample collection techniques are or will be included in pre-graduate courses. For example, such courses are already included in the curriculum of the University of Basel and will be included at the University of Geneva in the near future.

### 4.1. Post-Graduate Education in Travel Health

There is no mandatory post-graduate education related to travel health for community pharmacists in Switzerland. However, there are various continuous trainings that are available for pharmacists: e.g., the “Swiss Tropical and Public Health Institute—International Short Course on Travellers’ Health”, which gives relevant and updated information to assess travel-related health problems and to give preventive pre-travel advice, with a focus on tropical diseases, vaccination and prophylaxis; or the (in French) “Journée Romande de Médecine des Voyages”, which gives an annual update on various topics related to travel medicine. In addition, Swiss community pharmacists can participate in international trainings, such as the Conference of the International Society of Travel Medicine (CISTM), which is organized every two years [13].

### 4.2. Post-Graduate Education in Vaccination

Currently, each canton that authorizes community pharmacists to vaccinate requires pharmacists to hold an accreditation named (in French) “Certificat de formation complémentaire FPH (Foederatio Pharmaceutica Helvetiae) Vaccination et prélèvements sanguins”, that demonstrates their skills in vaccination, injection and blood samples techniques. This training lasts four and a half days. It includes theoretical and practical lessons, both with face-to-face and e-learning courses, followed by a complete “Adult basic life support and automated external defibrillation” (BLS AED). To renew his accreditation and maintain the authorization to vaccinate, each community pharmacist has to complete a minimum of one day of training related to vaccination at least every two years. Swiss or international continuous education related to travel medicine can be recognized as a part of this mandatory training [11].

## 5. Studies Related to Travel Health and Vaccination in Swiss Community Pharmacies

To our knowledge, no national study concerning activities in community pharmacies related to travels has been conducted in Switzerland.

Regarding vaccination, a national observational study, based on the voluntary reporting of influenza vaccination by community pharmacists registered on a paying web platform (www.vaccinationenpharmacie.ch), has been realized in the last influenza season (from 1 December 2017 to 31 January 2018) [14]. The results showed that 12,490 vaccinations were administered with written informed consent by accredited pharmacists active in 472 authorized pharmacies. A statistical extrapolation performed based on this study estimated that almost 20,000 influenza vaccinations were administered in authorized pharmacies over the same period [15]. The main limitation of this study is related to the fact that its results are based on the voluntary reporting of vaccinations on a paying web platform that is not systematically used. Hence, the total number of influenza vaccinations performed in Swiss community pharmacies over the same period is probably much larger.

Other studies related to vaccination in community pharmacies are currently underway at local levels (e.g., in the French-speaking canton of Vaud).

## 6. Discussion

Improving vaccination coverage by developing and implementing national vaccination plans is a major public health issue [16]. A global strategy has been conducted by health authorities to increase access to vaccination in the last several years in Switzerland [6]. Vaccination in Swiss community pharmacies has been identified as a mean to sustain this strategy, as it represents an option for adults who want to protect themselves, as well as their communities, and who do not have a referent GP or who do not want to visit their GP. This convenient access to vaccination is not only recognized in Switzerland and has already been implemented in several countries (e.g., Australia, Canada, Portugal, or the United States) [17]. Such strategies have to be adapted to each national context to be successful. In Switzerland, it considered the specificities regarding the different roles of health authorities at the national and cantonal levels. The national strategy recommended facilitating the contribution of community pharmacies to implement vaccination and defined the conditions and objectives for such involvement. Then, each canton determined the practical aspects to adapt the implementation to their particular context. Compared to a fully national strategy, this probably allowed to implement earlier vaccination in community pharmacies and to broaden the vaccines that can be administered by pharmacists in some cantons. This type of implementation process combining top-down and stepwise approaches (starting with influenza vaccination to demonstrate the competencies and impact of community pharmacists) can inspire other countries, especially federal ones. In addition, the parallel and complementarity development of pre- and post-graduate training programmes conducted by universities and professional associations of pharmacists seems to be an important element in such a strategy. Further developments in the continuous education of community pharmacists remain to be implemented. Indeed, community pharmacists are gaining more and more experience in vaccination. Hence, their needs related to the mandatory training required to renew their accreditation will certainly evolve. Advice related to travel health in community pharmacies has not yet been included in similar national and cantonal strategies. The increasing number of travels abroad by Swiss citizens [2] might lead to the development of new activities in community pharmacies, related to this specific public health issue, as in the United Kingdom [18]. Currently, this activity represents a good opportunity for strengthening travellers’ safety, beyond vaccination issues.

## 7. Conclusions

Top-down and stepwise strategies supported by education, accreditation and practice research activities showed to be effective to implement vaccination by Swiss community pharmacists. To finalize this implementation, continuous education should be adapted to meet new needs from community pharmacists experienced in vaccination. In addition, research should be supported to assess the effectiveness of community pharmacists, e.g., on vaccination coverage. Similar strategies should be conducted in other countries to involve community pharmacists in vaccination. In Switzerland, this could serve as a model to strengthen the role of community pharmacists in other public health developments.

## Figures and Tables

**Table 1:** Vaccinations authorized in community pharmacies, according to Swiss cantons (status in September 2018) [5].

Canton (Year of the Given Authorization to Perform Vaccination in Community Pharmacies)	Vaccinations Authorized in Community Pharmacies							
	Influenza	Tick-Borne Encephalitis	Hepatitis A	Hepatitis B	Hepatitis A and B	Measles, Mumps and Rubella	Human Papillomavirus	Diphtheria, Tetanus, and Pertussis
Lucerne (2017) Thurgau (2016)	+	+	+*	+*	+*	+*	+*	+*
Basel-Landschaft (2016)	+	+	+*	+*	+*	+*	-	-
Solothurn (2015)	+	+	+	+	+	+	-	-
Vaud (2016)	+	+	+*	+*	+*	+	-	-
Bern (2015), Graubünden (2016), Nidwalden (2017), Schaffhausen (2016), Schwyz (2016), Zug (2017), Zurich (2015)	+	+	+*	+*	+*	-	-	-
Fribourg (2015)	+	+	-	-	-	+	-	+
Basel-Stadt (2018)	+	+	+	+	+	-	-	-
Neuchâtel (2015)	+	+	-	-	-	+*	-	-
St. Gallen (2016)	+	+	-	-	-	-	-	-
Geneva (2016), Valais (2016), Jura (2016)	+	-	-	-	-	-	-	-

+: vaccination authorized in community pharmacy; +*: vaccination authorized in community pharmacy for the second dose, the first dose has to be administered by a physician; -: vaccination not authorized in community pharmacy.
